# Association analysis for resistance to *Striga hermonthica* in diverse tropical maize inbred lines

**DOI:** 10.1038/s41598-021-03566-4

**Published:** 2021-12-17

**Authors:** A. E. Stanley, A. Menkir, B. Ifie, A. A. Paterne, N. N. Unachukwu, S. Meseka, W. A. Mengesha, B. Bossey, O. Kwadwo, P. B. Tongoona, O. Oladejo, C. Sneller, M. Gedil

**Affiliations:** 1 West Africa Centre for Crop Improvement, University of Ghana, Legon, Ghana; 2 International Institute of Tropical Agriculture, Ibadan, Nigeria; 3 Ohio Agriculture Research and Development Center, Ohio State University, Wooster, OH USA

**Keywords:** Genetics, Plant sciences

## Abstract

*Striga hermonthica* is a widespread, destructive parasitic plant that causes substantial yield loss to maize productivity in sub-Saharan Africa. Under severe *Striga* infestation, yield losses can range from 60 to 100% resulting in abandonment of farmers’ lands. Diverse methods have been proposed for *Striga* management; however, host plant resistance is considered the most effective and affordable to small-scale famers. Thus, conducting a genome-wide association study to identify quantitative trait nucleotides controlling *S. hermonthica* resistance and mining of relevant candidate genes will expedite the improvement of *Striga* resistance breeding through marker-assisted breeding. For this study, 150 diverse maize inbred lines were evaluated under *Striga* infested and non-infested conditions for two years and genotyped using the genotyping-by-sequencing platform. Heritability estimates of *Striga* damage ratings, emerged *Striga* plants and grain yield, hereafter referred to as *Striga* resistance-related traits, were high under *Striga* infested condition. The mixed linear model (MLM) identified thirty SNPs associated with the three *Striga* resistance-related traits based on the multi-locus approaches (mrMLM, FASTmrMLM, FASTmrEMMA and pLARmEB). These SNPs explained up to 14% of the total phenotypic variation. Under non-infested condition, four SNPs were associated with grain yield, and these SNPs explained up to 17% of the total phenotypic variation. Gene annotation of significant SNPs identified candidate genes (Leucine-rich repeats, putative disease resistance protein and VQ proteins) with functions related to plant growth, development, and defense mechanisms. The marker-effect prediction was able to identify alleles responsible for predicting high yield and low *Striga* damage rating in the breeding panel. This study provides valuable insight for marker validation and deployment for *Striga* resistance breeding in maize.

## Introduction

Maize (*Zea mays* L.) is an important cereal that plays a crucial role in alleviating food insecurity in sub-Saharan Africa (SSA) due to its high yield potential, ease in processing and low cost^1^. However, its production is constantly hampered by a plethora of biotic stresses, including the parasitic weed *Striga*. Among the numerous *Striga* species endemic to Africa, *Striga hermonthica* (Del.) Benth is the most destructive and widespread, affecting cereals, including maize and sorghum (*Sorghum bicolor L.*)^2^. Yield losses attributed to *Striga* infestation in maize range from 60 to 100% under severe field infestation, especially in marginal production areas where smallholder farmers cannot afford high inputs and other control measures^3^.

*Striga hermonthica* is an obligate root hemiparasite, which depends on its host for survival, notwithstanding its photosynthetic capacity after emergence from the soil^4^. The parasite’s lifecycle is intimately associated with its host to ensure its survival^5^. The interaction between the parasitic plant and its host is initiated immediately a chemical compound known as strigolactone is released from the host plant. This chemical compound stimulates the germination of *Striga* seeds. Once the *Striga* seeds germinates, it establishes a connection with the roots of its host, extracting water, carbon and essential nutrients for its growth. The parasitic plant inflicts more damage on its host underground before its emergence from the soil, and this damage is accentuated in areas affected by sub-optimal soil fertility and recurrent drought^6^. *Striga hermonthica* parasitism is characterized by chlorosis, firing of leaves around margins, wilting, stunting, poorly filled ears, and death under severe infestation^7^. *Striga* resistance is a complex quantitative trait controlled by multiple genes/polygenes, and it is highly affected by environmental factors^8^.

Several control methods have been proposed for *Striga* management, including host plant resistance, cultural, chemical and manual control options. However, integrated *Striga* management approach is considered the most economical and affordable for small-scale farmers who cannot afford high inputs control options. The approach involves the combination of two or more control options. Host plant resistance is considered a cost effective, environmental feasible and affordable option for smallholder farmers. It is also an essential component of any successful integrated approach for controlling *Striga* parasitism. Several studies have shown progress in breeding for natural genetic resistance to *Striga* in maize^5^. In addition, extensive research has been done to map quantitative trait loci (QTLs) for *Striga* resistance in maize using molecular markers. QTL mapping and genome-wide association study (GWAS) are two methods widely used to discover genetic loci controlling complex traits. Quantitative trait loci (QTLs) associated with *Striga hermonthica* have been identified in maize using QTL mapping approach^9–11^. Badu-Apraku et al.^9^ identified 12 QTLs associated with four *Striga* resistance/tolerance traits in maize and these QTLs explained 3.2 to 34.9% of the phenotypic variation. However, the QTL mapping approach have several limitations, for example, it has limited allelic diversity, and limited mapping resolution due to limited recombination events^12^. On the other hand, GWAS explores ancestral recombination in natural genetically diverse population to dissect complex traits^13^. GWAS is an improvement over QTL mapping in that it improves the resolution of QTLs due to accumulated meiotic events and reduces the time taken in developing mapping populations^14^. GWAS is a powerful tool for detecting QTLs associated with important complex quantitative traits, as well as predicting or identifying causative genes^15^.

Many statistical models have been developed to improve the power of identifying QTNs with the GWAS approach. The single-locus mixed-linear model (MLM) is the most common method used for GWAS. The method uses several algorithms such as the compressed MLM^16^, enriched MLM^17^, however, all these models perform single dimensional genome scan and require multiple correction. These models also have major limitations in mapping QTNs with small effects. Wang et al.^18^ proposed a new model based on multi-locus random-SNP-effect MLM (mrMLM). The methods include polygenic-background control-based least angle regression plus empirical Bayes (pLARmEB), fast multi-locus random-SNP-effect efficient mixed model association (FASTmrEMMA), iterative-sure independence screening expectation–maximization (EM)-Bayesian LASSO (ISIS EM-BLASSO) and fast multi-locus random-SNP-effect mixed linear model (FASTmrMLM)^18–22^. These methods can effectively detect small-effect QTNs and improve the efficiency and accuracy of GWAS. Recently, few studies have implemented the above GWAS methods to detect important loci controlling different traits in maize^23^.

Genome-wide association study (GWAS) for *S. hermonthica* resistance has been conducted in maize. Adewale et al.^24^ identified 24 SNPs that were significantly associated with four *Striga* resistance-related traits in early maturing maize inbred lines using compressed MLM, these lines captured only the genetic variation existing in the extra early and early maturing maize germplasm developed at IITA. However, genomic regions governing *Striga* resistance in many intermediate and late-maturing maize inbred lines with consistent expression of polygenic resistance to *S. hermonthica* have not been identified. This germplasm offers an excellent resource for discovering functional genes underlying the genetic variation in the *Striga* resistance-related traits. This study was thus conducted; to evaluate diverse intermediate and late maturing maize inbred line for *Striga* resistance under *Striga* infested and non-infested conditions and identify genomic regions and candidate genes related to *Striga* resistance.

## Results

### Phenotypic diversity

In the combined analyses of variance (ANOVA), environments and lines had significant (*p* < 0.001) effects on the three *Striga* resistance-related traits under *Striga* infested conditions and for grain yield under non-infested condition (Tables 1 and 2). The line x environment interaction mean squares were also significant for most of the traits measured under the two conditions.

**Table 1:** Mean squares from the combined ANOVA for traits recorded under *Striga* infestation across four environments.

Source	DF	Yield (kg/ha)	*Striga* damage score at 8 WAP (1–9)	*Striga* damage score at 10 WAP (1–9)	Emerged *Striga* count at 8 WAP	Emerged *Striga* count at 10 WAP
Env (E)	3	104128033**	172.75^§^	445.94^§^	5792.54^§^	39,237.32^§^
Rep(Env)	4	2831059**	1.26 ns	4.05 ns	91.92 ns	301.57 ns
Blk(Rep*E)	112	524846***	1.11^§^	1.33***	451.34^§^	1112.03^§^
Lines (L)	149	2559095^§^	7.00^§^	7.81^§^	1528.28^§^	2435.87^§^
L x E	446	483792^§^	1.18^§^	1.40^§^	268.44**	530.09**
Error	483	321643	0.63	0.83	204.33	416.2
CV (%)		35.95	19.62	15.45	61.34	58.53

*, **, ***, ^§^Significant at *p* ≤ 0.05, *p* ≤ 0.01, *p* ≤ 0.001 and 0.0001 levels, respectively, ns = not significant.

Blk, Block; Env, Environment; Rep, Replication; CV, Coefficient of Variation; WAP, weeks after planting.

**Table 2:** Mean squares from the combined ANOVA for traits recorded under non-infested conditions across four environments.

Source	DF	Yield (kg/ha)
Env (E)	3	175723422.1^§^
Rep(Env)	4	2428705***
Blk(Rep*Env)	112	1281736.9^§^
Lines (L)	149	3287995^§^
L x E	446	732612.7^§^
Error	483	470085
CV (%)		32.73

*, **, ***, ^§^Significant at *p* ≤ 0.05, *p* ≤ 0.01, *p* ≤ 0.001 and 0.0001 levels, respectively, ns = not significant.

Blk, Block; Env, Environment; Rep, Replication; CV, Coefficient of Variation; WAP, weeks after planting.

Further assessment of the line x environment interaction using rank correlation analyses between pairs of environments found highly significant (*p* < 0.0001) correlations for the *Striga* resistance-related traits (Supplementary Table S1). The broad-sense heritability estimates for the *Striga* resistance-related traits varied from 81 to 85% (Supplementary Table S2).

Grain yield under *Striga* infestation varied from 13 to 3299 kg/ha with an average of 1580 kg/ha, while grain yield under non-infested condition varied from 706 to 4171 kg/ha with an average of 2098 kg/ha (Supplementary Table S2). Relative to the non-infested condition, the resistant benchmark (9450) suffered 46% yield loss, whereas the susceptible benchmark (5057) suffered 77% yield loss indicating the inbred lines used in this study were exposed to severe *Striga* infection. In addition, 86 maize inbred lines supported significantly fewer emerged *Striga* plants at 8 and 10 weeks after planting (WAP). These lines, on average, suffered 19% yield reduction relative to the non-infested conditions, produced significantly higher grain yields than the resistant benchmark (9450), and were categorized as resistant lines. In contrast, 44 inbred lines supported as many *Striga* plants as the susceptible benchmark (5057) but produced significantly higher grain yields than the susceptible line, and were categorized as tolerant. The remaining 20 inbred lines, supported as many *Striga* plants as the susceptible benchmark and did not differ significantly from the susceptible line in their grain yields, and were regarded as susceptible. All paired traits showed statistically significant differences at *p*-value < 0.01. A negative correlation was observed between grain yield and *Striga* damage rating and emerged *Striga* plants at 8 and 10 WAP. However, there was a positive correlation between *Striga* damage rating and emerged *Striga* plants at 8 and 10 WAP (Supplementary Fig. S1).

### Genotyping

#### Population structure and linkage disequilibrium

**Figure 1:**
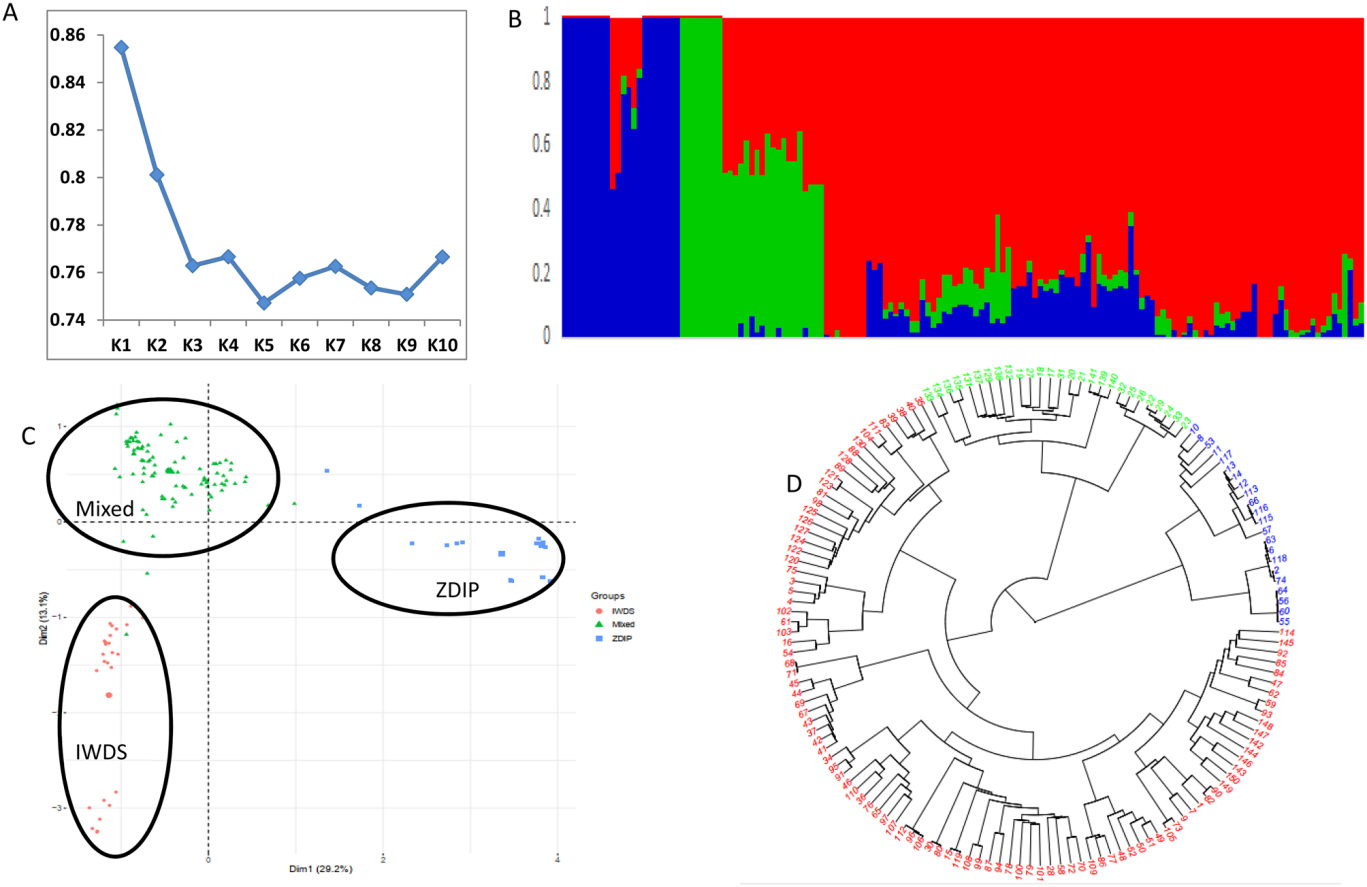
(**A**) Cross-validation plot showing the optimal number of clusters. (**B**) Population structure plot of the inbred lines (k = 3). C) Principal component analysis based on 150 maize inbred lines using the 16,735 SNP markers. D) Phylogenetic tree showing the genetic relationship among 150 diverse maize inbred lines.

For the genotypic analysis, 16,735 SNPs distributed across the ten maize chromosomes were identified after the quality control process. A minimum of 1208 SNPs (7.2%) were mapped on chromosome 10, whereas a maximum of 2532 SNPs (15.2%) were mapped on chromosome 1. The Admixture analysis using tenfold cross-validation from *k* = 1 to *k* = 10 showed a sharp elbow at *k* = 3, indicating the inbred lines can be grouped into three subgroups (Fig. 1A,B). The principal component analysis (PCA) also grouped the inbred lines into three subgroups and this is consistent with the Admixture results. A scree plot generated to visualize the fraction of variance represented by each of the ten principal components showed that two (PC1 and PC2) explained the largest proportion (42.3%) of the total variance (Fig. 1C). Furthermore, the phylogeny tree clustering also grouped the inbred lines into three distinct subgroups; 22, 27 and 101, derived from ZDIP, IWDS and Mixed groups, respectively (Fig. 1D). The assignment of the inbred lines into the three subgroup based on the phylogeny tree were in good agreement (98%) with those revealed by PCA The inbred lines were grouped based on their genetic background/pedigree and maturity. The LD estimates (*r*^2^) showed a slight increasing and then consistent pattern of LD decay was observed with an increase in the physical distance of SNP markers mapped on the 10 chromosomes (Supplementary Fig. S2). The average linkage disequilibrium decay varied from 2.73 kb on chromosome 6 to 3.68 kb on chromosome 8 (Supplementary Table S3).

#### Genome-wide association analysis

**Figure 2:**
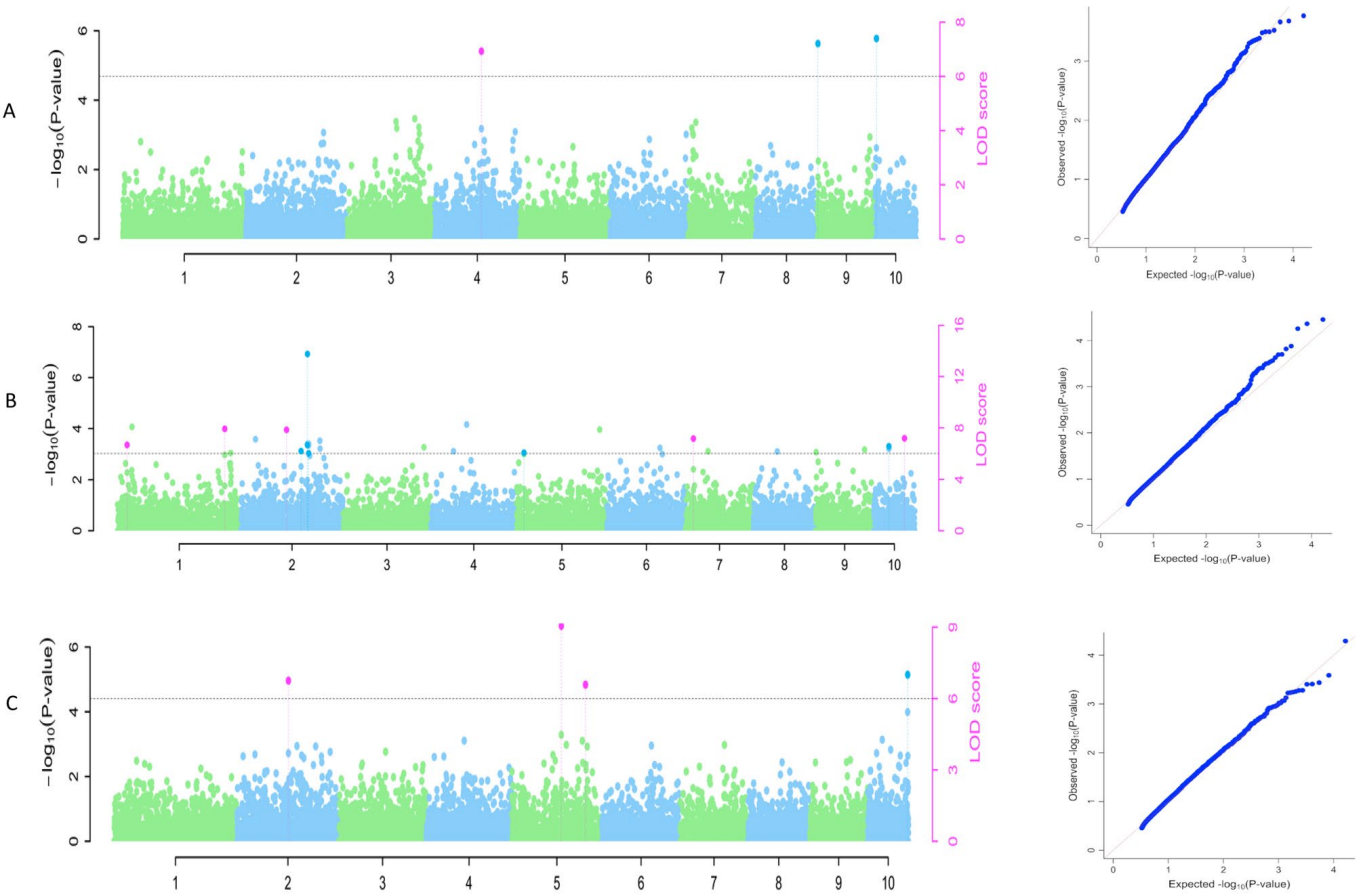
Manhattan plot indicating SNPs associated with (**A**) Grain yield, (**B**) *Striga* damage score at 10 WAP (**C**) Emerged *Striga* plants at 10 WAP. The graph refers to the quantile–quantile (Q-Q) plot of the P-values observed and expected from the association analysis under *Striga* infestation**.**

The GWAS multiple-locus models used in this study identified 30 significant SNPs that were significantly associated with the three *Striga* resistance-related traits. These SNPs were distributed on all maize chromosomes but chromosome 8. The highest number of SNPs was found on Chromosome 1, and the least on chromosomes 3, 6 and 7 (Table 3). The results of the Manhattan plot and the quantile–quantile plots revealed reasonable data adjustment and a few significant SNPs above the interval of the expected values of the null hypothesis (Fig. 2). This study employed four multi-locus methods, mrMLM, FASTmrMLM, FASTmrEMMA, and pLARmEB to perform comprehensive GWA mapping in our diversity panel. Among the four methods mrMLM identified the highest number of SNPs (19) while, FASTmrEMMA identified the least (5). In addition, FASTmrMLM identified the most codetected SNPs among the four GWAS models used. Four SNPs were associated with grain yield under *Striga* infested condition. These SNPs are located on three chromosomes, and each SNP explained between 3.21 to 13.36% of the phenotypic variation. One of the SNPs (S4_164335765) associated with grain yield was detected by two GWAS multi-locus methods (FASTmrMLM and mrMLM) and they explained 6.7 and 13.4% of the phenotypic variation. The LOD score of the significant SNPs ranged from 7.18 (S4_164335765) to 7.39 (S10_1784894).

**Table 3:** Significant SNPs identified under *Striga* infested condition.

Trait name	SNP	Chr	Position (bp)	LOD	PVE (%)	Favorable allele	Methods
YLDIN_G	S4_164335765	4	164335765	7.18	13.36	A	1, 2
	S9_1994432	9	1994432	7.21	3.21	G	4
	S10_1784894	10	1784894	7.39	3.58	C	4
Striga damage rating at 8 WAP	S1_18512344	1	18512344	10.76	14.19	T	1
	S2_14081759	2	14081759	8.26	7.89	A	1
	S2_160791711	2	160791711	7.28	3.35	A	1
	S2_219240910	2	219240910	11.31	8.05	G	3
	S4_160459526	4	160459526	6.07	3.83	C	1
	S5_70442824	5	70442824	6.76	3.22	C	2
	S5_216138908	5	216138908	6.81	6.67	C	4
	S6_25428338	6	25428338	6.58	0.142	A	4
	S10_25285761	10	25285761	11.87	6.57	G	2
	S10_2743583	10	2743583	9.86	0.24	C	4
	S10_68324912	10	68324912	11.79	1.32	C	4
Striga damage rating at 10 WAP	S1_284192573	1	284192573	8.54	10.40	G	1, 2, 3
	S1_10576247	1	10576247	6.45	10.00	A	1, 2
	S2_190557148	2	190557148	6.02	4.13	T	1
	S2_99667127	2	99667127	7.82	4.82	A	1, 2
	S2_160791711	2	160791711	6.18	4.37	A	3
	S2_188120710	2	188120710	13.76	6.47	A	4
	S5_5623740	5	5623740	6.06	13.97	C	1
	S7_10795659	7	10795659	6.71	7.99	A	1, 2
	S10_25285761	10	25285761	6.56	5.04	G	3
Emerged Striga plants at 8 WAP	S1_298950342	1	298950342	6.15	6.58	A	1
	S2_135038935	2	135038935	6.67	6.66	G	3, 4
	S2_208978140	2	208978140	6.82	6.97	C	3, 4
	S3_74335447	3	74335447	6.21	5.54	A	1
	S5_148751913	5	148751913	6.41	6.21	A	3, 4
	S9_7727167	9	7727167	8.32	11.51	G	1, 2
	S10_125571525	10	125571525	9.28	13.08	G	1, 2, 4
	S10_90133328	10	90133328	7.75	13.58	T	1, 2, 4
Emerged Striga plants at 10 WAP	S2_135038935	2	135038935	6.83	12.22	G	1, 2
	S5_148751913	5	148751913	8.65	7.91	A	1, 2
	S5_204969099	5	204969099	6.93	10.22	T	1, 2
	S10_125571525	10	125571525	7.00	6.33	G	4

Methods: Numbers 1 to 4 represents different GWAS methods: 1: mrMLM; 2: FASTmrMLM; 3: FASTmrEMMA; 4 pLARmEB Chr, Chromosome; YLDIN_G, Grain yield; WAP, weeks after planting.

Eleven SNPs were associated with *Striga* damage rating at 8 WAP. These SNPs are located on seven chromosomes and the proportion of phenotypic variation explained by each SNP ranged from 0.14 to 14.19%. The LOD values of the identified SNPs ranged from 6.06 (S4_160459526) to 11.80 (S10_68324912). Nine SNPs were associated with *Striga* damage rating at 10 WAP. These SNPs were located on five chromosomes, and they explained 4.13 to 11.60% of the phenotypic variation. Four SNPs (S1_284192573, S1_10576247, S2_99667127, and S7_10795659) associated with *Striga* damage rating at 10 WAP were detected by two or more of the GWAS multi-locus (mrMLM, and FASTmrMLM, and FASTmrEMMA) methods.

The LOD values of the identified SNPs ranged from 6.02 (S2_190557148) to 13.76 (S2_188120710). In general, two SNPs (S2_160791711 and S10_25285761) were simultaneously associated with *Striga* damage rating at 8 and 10 WAP.

Eight SNPs were associated with emerged *Striga* plants at 8 WAP. These SNPs are located on five chromosomes and the proportion of phenotypic variation explained by each SNP ranged from 5.03 to 13.58%. Five SNPs (S2_208978140, S2_135038935, S5_148751913, S9_7727167, and S10_90133328) associated with emerged *Striga* plants at 8 WAP were detected by two or more of the GWAS multi-locus (mrMLM, FASTmrMLM, FASTmrEMMA, and pLARmEB) methods. The LOD values of the identified SNPs ranged from 6.14 (S1_298950342) to 9.28 (S10_125571525). Four SNPs were associated with emerged *Striga* damage plants at 10 WAP. These SNPs are located on three chromosomes and the proportion of phenotypic variation explained by each SNP ranged from 6.33 to 12.21%. Three SNPs (S2_135038935, S5_148751913, and S5_204969099) associated with emerged *Striga* plants at 10 WAP were detected by two GWAS multi-locus (mrMLM, and FASTmrMLM) methods. The LOD values of the identified SNPs ranged from 6.83 (S2_135038935) to 8.65 (S5_148751913). In general, three SNPs (S2_135038935, S5_148751913, and S10_12557152) were simultaneously associated with emerged *Striga* plants at 8 and 10 WAP.

Under non-infested conditions, four SNPs were associated with grain yield (Table 4). These SNPs are located on three chromosomes and the proportion of phenotypic variation explained by each SNP ranged from 5.63 to 17.40%. Furthermore, one of the SNPs on chromosome 1 (S4_189154251) was detected by two of the GWAS multi-locus methods (mrMLM, and FASTmrMLM). The LOD score of these SNPs ranged from 6.52 (S1_26517984) to 9.72 (S4_189154251).

**Table 4:** Significant SNPs identified under non-infested condition.

Trait name	SNP	Chr	Position (bp)	LOD score	PVE (%)	Favorable allele	Methods
YLDUN_G	S1_14334790	1	14334790	7.0841	10.2684	C	1
	S1_26517984	1	26517984	6.5272	5.6321	G	2
	S4_189154251	4	189154251	9.7264	17.4015	A	1, 2
	S8_17462112	8	17462112	6.5008	7.0482	G	4

Methods: Numbers 1 to 4 represents different GWAS methods: 1: mrMLM; 2: FASTmrMLM; 4 pLARmEB Chr = Chromosome, YLDIN_G = Grain yield, WAP = weeks after planting.

#### Markers effect prediction

**Figure 3:**
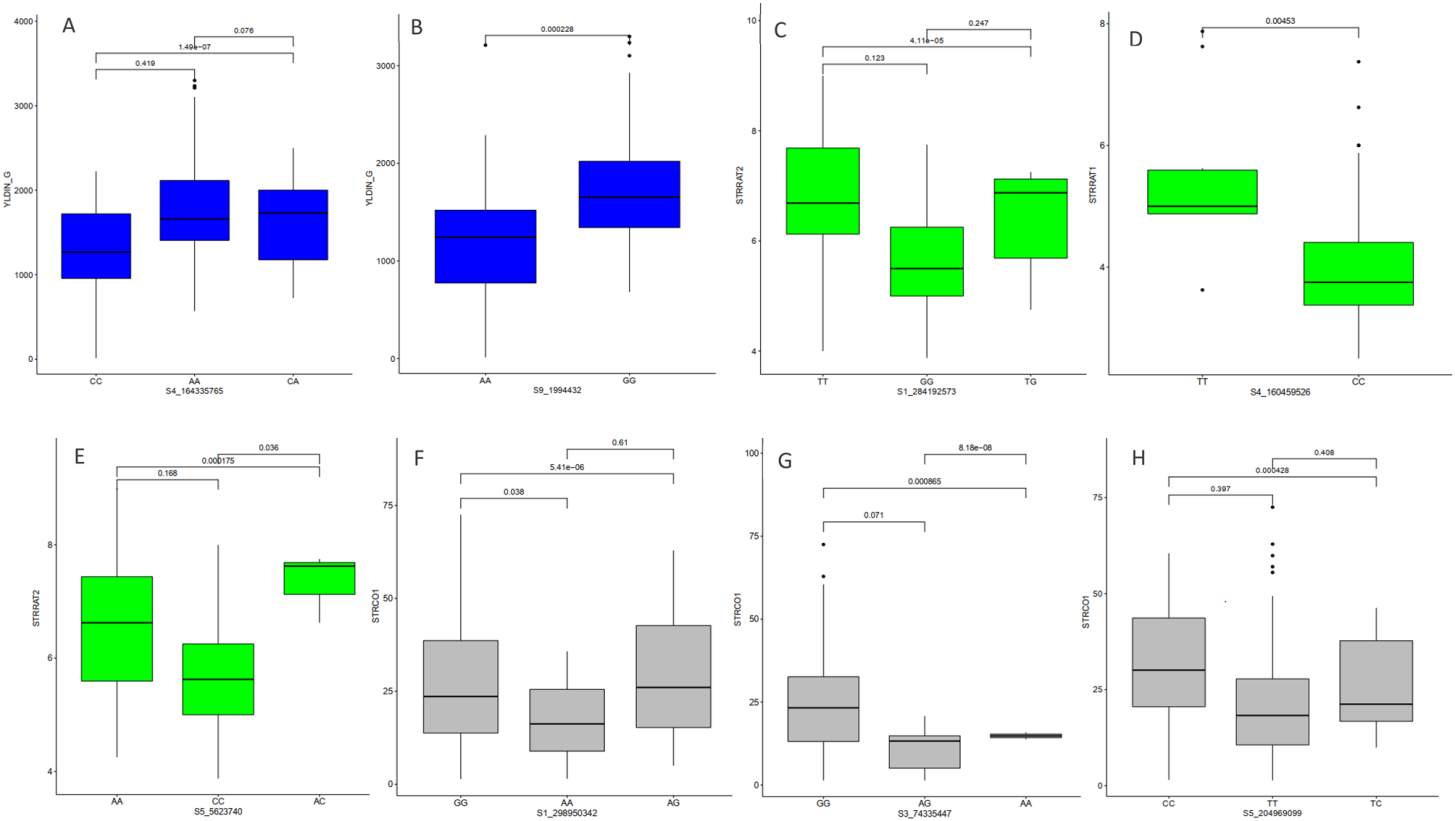
Allelic effects of haplotype blocks associated with Grain yield (**A**,**B**) blue colour, *Striga* damage ratings (**C**–**E**) green colour, emerged *Striga* plants (**F**,**G**) gray colour under *Striga* infestation.

The frequencies and marker prediction effects of various alleles associated with the three *Striga* resistance-related traits are presented in Table 5. Two of the SNPs (S4_164335765 and S9_1994432) associated with grain yield under *Striga* infestation displayed high segregation among the inbred lines. For SNPs on chromosome 4, alleles AA and CA were associated with genotypes with higher grain yield, while alleles CC were associated with lower grain yield. For the second SNP on chromosome 9, alleles GG were associated with genotypes with higher grain yield, while alleles AA were associated with lower grain yield (Fig. 3). For *Striga* damage ratings at 8 and 10 WAP, three SNPs (S1_284192573, S4_160459526, and S5_5623740) displayed high segregation among the inbred lines. For two of the SNPs on chromosomes 1 and 4, alleles TT and TG were associated with high *Striga* damage ratings, while alleles GG and CC were associated with low *Striga* damage rating. For the SNP on chromosome 5, alleles AA and AC were associated with high *Striga* damage ratings, while alleles CC were associated with low *Striga* damage ratings at 8 and 10 WAP (Fig. 3). For emerged *Striga* plants, four SNPs (S1_298950342, S3_74335447, and S5_204969099) displayed high segregation among the inbred lines. For the two SNPs on chromosomes 1 and 3, variants GG and AG supported the emergence of more *Striga* plants whereas, alleles AA supported little emerged *Striga* plants. For the SNP on chromosome 5, variants CC supported the emergence of more *Striga* plants, while alleles AA and AC supported little emerged *Striga* plants (Fig. 3).

**Table 5:** Frequencies and marker prediction effects of various alleles associated with the *Striga* resistance-related traits.

Trait	Marker name	Allele1	Allele2	Sequence	Frequency	Adjusted probability	Adjusted significance
Grain yield	S4_164335765	AA	AC	AAAC	0.34	4.19E-01	ns
		AA	CC	AACC	0.47	4.47E-07	****
		AC	CC	ACCC	0.21	1.52E-01	ns
	Ch9_1994432	AA	GG	AAGG	1.00		***
*Striga* damage rating	Ch1_284192573	GG	GT	GGGT	0.42	2.46E-01	ns
		GG	TT	GGTT	0.48	1.23E-04	***
		GT	TT	GTTT	0.10	2.47E-01	ns
	Ch4_160459526	CC	TT	CCTT	1.00		**
	Ch5_5623740	AA	AC	AAAC	0.12	1.68E-01	ns
		AA	CC	AACC	0.49	5.25E-04	***
		AC	CC	ACCC	0.39	7.20E-02	ns
	Ch5_216138908	AA	CC	AACC	1.00		***
Emerged *Striga* plants	Ch1_298950342	AA	AG	AAAG	0.23	7.50E-02	ns
		AA	GG	AAGG	0.46	1.62E-05	****
		AG	GG	AGGG	0.31	6.10E-01	ns
	Ch3_74335447	AA	AG	AAAG	0.06	7.10E-02	ns
		AA	GG	AAGG	0.45	2.00E-03	**
		AG	GG	AGGG	0.49	2.45E-07	****
	Ch5_148751913	AA	AC	AAAC	0.36	3.29E-01	ns
		AA	CC	AACC	0.16	4.60E-02	*
		AC	CC	ACCC	0.48	4.60E-02	*
	Ch5_204969099	CC	CT	CCCT	0.13	7.04E-01	ns
		CC	TT	CCTT	0.48	4.56E-04	***
		TT	TC	TTTC	0.39	7.04E-01	ns

### Identifying putative genes

According to the genomic information of B73 Ref Gen_V4, thirty-one putative genes/proteins, including two uncharacterized proteins were found in the intervals adjacent to the significant SNPs detected for the three *Striga hermonthica* resistance-related traits (Table 6). Remarkably, most of the gene models identified encode transcription factors, disease resistance proteins, zinc-finger domain proteins, leucine-rich repeats protein kinase and some pathogenesis-related proteins. Most of the identified genes were located on chromosomes bins 1.10, 2.05, 2.06, 3.04, 6.01, 7.01. 9.01, 10.00. 10.01, and 10.03.

**Table 6:** Significant SNPs associated with the *Striga* resistance-related traits and putative genes identified for the 150 maize inbred lines.

Trait	SNP	Position (bp)	Gene ID	Putative Gene
Grain yield	S4_164335765	164335765	GRMZM2G157836; GRMZM5G881641	adenylyltransferase and sulfurtransferase (MOCS3 2)
	S9_1994432	1994432	GRMZM2G406758; GRMZM2G110289	U-box domain-containing protein 39; NLR family CARD domain-containing protein 3
	S10_1784894	1784894	GRMZM2G180262	VQ
*Striga* damage score at 8 and 10 WAP	S1_18512344	18512344	GRMZM2G024099	Aspartyl protease AED3
	S1_284192573	284192573	GRMZM2G351582	ZPR1 zinc-finger domain protein (uaz7c01h10)
	S1_10576247	10576247	GRMZM2G028521	citrate transporter family protein (pco091082)
	S2_14081759	14081759	GRMZM2G092128	E3 ubiquitin-protein ligase PUB23
	S2_160791711	160791711	GRMZM2G102242	meiotic nuclear division protein 1 homolog
	S2_190557148	190557148	GRMZM2G414252	bHLH transcription factor (bHLH20)
	S2_99667127	99667127	GRMZM2G171830	Protein TIFY 10B
	S2_219240910	219240910	GRMZM2G162781	putative leucine-rich repeat protein kinase family protein
	S4_160459526	160459526	GRMZM2G081285	RING-H2 finger protein ATL1R
	S5_5623740	5623740	GRMZM2G112548	transcription factor JUNGBRUNNEN 1
	S5_216138908	216138908	GRMZM2G113418	glutaredoxin 2
	S5_70442824	70442824	GRMZM2G035073	putative cytochrome P450 superfamily protein
	S6_25428338	25428338	GRMZM5G832409	knotted related homeobox 5 (lg4b)
	S7_10795659	10795659	GRMZM2G162382	Transcription factor bHLH7
	S10_25285761	25285761	GRMZM2G300965; GRMZM2G300969	uncharacterized LOC100381459; leucine-rich repeat extensin-like protein 3
	S10_2743583	2743583	GRMZM5G873586; GRMZM2G356817	Disease resistance protein RPM1; putative disease resistance RPP13-like protein 1
	S10_68324912	68324912	GRMZM2G364748	Xyloglucan endotransglycosylase (gpm554)
Emerged *Striga* plants at 8 and 10 WAP	S1_298950342	298950342	GRMZM2G017470	Dof zinc finger protein
	S2_135038935	135038935	GRMZM2G088778	Protein ACCELERATED CELL DEATH 6
	S2_208978140	208978140	GRMZM2G179505	hydrolase/ protein serine/threonine phosphatase
	S3_74335447	74335447	GRMZM2G701566	uncharacterized LOC100382572
	S5_148751913	148751913	GRMZM2G129543	peroxidase 70
	S5_204969099	204969099	GRMZM5G823157	probable WRKY transcription factor 14
	S9_7727167	7727167	GRMZM2G033413	bZIP transcription factor 46
	S10_125571525	125571525	GRMZM2G006948	hapless 8
	S10_90133328	90133328	GRMZM2G063575	Pentatricopeptide repeat-containing protein

Five gene models were identified around three SNPs associated with grain yield under *Striga* infestation. Two gene models each were found on chromosomes 4 and 9 and they encode adenylyltransferase, sulfurtransferase (MOCS3 2), U-box domain-containing protein 39, and NLR family CARD domain-containing protein 3. The remaining gene model on chromosome 10 encodes VQ proteins. These putative genes/proteins are mainly involved in developmental processes including responses to biotic and abiotic stress, seed development and photo-morphogenesis. The LD heat-map of significant SNPs (S9_1994432 and S10_1784894) identified on chromosomes 9 and 10 were highly correlated (0.5 to 0.8) with regions adjacent to the identified putative genes (U-box domain-containing protein 39, NLR family CARD domain-containing protein 3 and VQ proteins) (Supplementary Fig. S3).

Nineteen gene models were identified around seventeen SNPs associated with *Striga* damage ratings at 8 and 10 WAP. Two gene models each were associated with SNPs S10_25285761 and S10_2743583 located on chromosome 10. These gene models encode leucine-rich repeat extension-like protein, disease resistance protein RPM1, disease resistance RPP13-like protein1 and an uncharacterized protein. Other genes models associated with *Striga* damage ratings encode putative cytochrome P450 superfamily protein, xyloglucan endotransglycosylase, basic helix-loop-helix (bHLH7 and bHLH20) transcription factors, knotted related homeobox 5, ubiquitin-protein ligase, and zinc-finger domain proteins. Most of these genes/proteins identified are involved in different development and plant defense mechanism. Plant resistance genes allow plants recognize the presence of specific pathogens and initiate defense responses. The LD heat-map of significant SNPs (S5_70442824, S7_10795659 and S10_25285761) identified on chromosomes 5, 7 and 10 were highly correlated (0.5 to 0.8) with regions adjacent to the identified putative genes (putative cytochrome P450 superfamily protein, Transcription factor bHLH7, uncharacterized LOC100381459 and leucine-rich repeat extensin-like protein 3) (Supplementary Fig. S4).

Nine gene models were identified around nine SNPs associated with emerged *Striga* plants at 8 and 10 WAP. These gene models encode Dof zinc finger protein, protein accelerated cell death 6, peroxidase 70, hapless, basic-domain leucine-zipper (bZIP46) and WRKY14 transcription factor. Transcription factors are usually involved in diverse plant processes including, growth, development and stress signaling. In addition, protein accelerated cell death is a positive regulator of programmed cell death and it is a mechanism used by plants for defense against pathogen infection. The LD heat-map of significant SNPs (S3_74335447 and S9_7727167) identified on chromosomes 3 and 9 were highly correlated (0.4 to 0.8) with regions adjacent to the putative genes (uncharacterized LOC100382572, bZIP transcription factor 46) (Supplementary Fig S5).

Under non-infested conditions, six gene models were identified around four SNPs associated with grain yield (Table 7). Most of the identified genes were located on chromosomes bins 1.02, 4.08 and 8.02. These gene models encode IQ domain-containing protein IQM5, uncharacterized protein LOC100277298, WRI1 transcription factor 2, ENTH/VHS family protein, Replication protein A 70 kDa DNA-binding and Photosystem I reaction center subunit XI. IQ-domain proteins are common in land plants and they are known for critical roles in host defense, cell shaping and drought resistance.

**Table 7:** Quantitative trait nucleotides (QTNs) associated with grain yield under non-infested condition and putative genes identified for the 150 maize inbred lines.

Trait	SNP	Position	Gene ID	Putative Gene
Grain yield	S1_14334790	14334790	GRMZM2G424020, GRMZM2G464363	IQ domain-containing protein IQM5, uncharacterized protein LOC100277298
	S4_189154251	189154251	GRMZM2G174834, GRMZM2G174938	WRI1 transcription factor 2, ENTH/VHS family protein
	S1_26517984	26517984	GRMZM2G098714	Replication protein A 70 kDa DNA-binding
	S8_17462112	17462112	GRMZM2G026015	Photosystem 1 reaction center subunit XI

## Discussion

The marked reduction in grain yield observed in the resistant and susceptible benchmark indicates the occurrence of severe parasite infestation across the test environments, eliciting significant differences in resistance or susceptibility reactions among the inbred lines. The diversity panel used in our study displayed considerable phenotypic variation for the three *Striga* resistance-related traits recorded under *Striga* infestation, and this is consistent with the findings in other studies^25^. The significant line x environment interaction observed for traits measured under *Striga* infestation can be attributed to varying seasonal factors, soil pH, and nutrient levels^26^. Also, the significant rank correlations among environments for the major *Striga* resistance-related traits indicates that the lines maintained consistent resistance or susceptibility reactions to *Striga* seeds collected from different locations and years. More than 55% of the lines evaluated in this study were resistant to *S. hermonthica,* and this is due to the severe selection pressure imposed by the breeders during the development of these inbred lines from diverse source populations.

Heritability estimates were high for the *Striga* resistance-related traits, indicating the predominant role of genetic factors for these traits. Traits with high heritability increase the power of detecting SNPs in an association panel and thus allow the identification of a true association between a marker and a putative gene^15^. The high heritability estimates observed for *Striga* damage ratings and emerged *Striga* plants in this study is consistent is consistent with the results reported by Najar et al.^27^ and Shayanowako et al.^25^. These findings, however, differ from those of Badu-Apraku et al.^28^, who recorded low heritability estimates (h^2^ < 50) for emerged *Striga* plants and *Striga* damage ratings. .

The efficiency of association mapping largely depends on population size and population structure, which infers the ancestry of lines based on their genotypic information^29^. The diversity panel used in this study are inbred lines derived from broad-based populations containing tropical and temperate germplasm, backcrosses containing *Z. diploperennis* adapted to tropical environments, and some lines that are tolerant to drought^30^. The two complementary approach used to infer the population structure grouped the inbred lines into three subpopulations based on their genetic backgrounds/pedigree and maturity information. It is worth noting that there was high agreement in the assignment of the inbred lines into the three subgroups based on the two approaches (Admixture and PCA). The phylogeny tree also grouped the inbred lines into three subgroups.

LD is an important factor that determines the power of marker-trait association analysis. In this study, more than 60% of the SNP pairs across the genome exhibit LD at r^2^ > 0.1. In addition, the high r^2^ value observed on chromosomes 4 and 8 in this study can be attributed to fewer recombination events on these chromosomes; this is consistent with the findings of Thirunavukkarasu et al.^31^ and Dinesh et al.^32^, who reported high r^2^ value on chromosome 4 and 8 of maize. Reports have shown that the effectiveness of recombination is limited by the high level of homozygosity^33^. In this study, faster decay of LD with increasing distance between markers was observed, which agrees with Doa et al.^34^ and Dinesh et al.^32^.

Several studies have been conducted to dissect the genetic architecture of *Striga* resistance in maize, and many QTLs associated with *Striga* resistance have been detected using bi-parental populations^9–11^. Badu-Apraku et al.^10^ identified 116 QTLs associated with four *Striga* resistance-related traits (grain yield, *Striga* damage ratings, ear aspect and emerged *Striga* plants) using bi-parental (F_2:3_) population derived from two early maturing maize lines. In another study, Badu-Apraku et al.^9^ identified 14 QTLs that were associated with three *Striga* resistance-related traits (grain yield, ears per plant and *Striga* damage rating at 10 WAP). However, QTL mapping only exploits only a small fraction of available genetic diversity and exhibits limited capacity to detect polygenic resistance^12^. However, only a few GWAS has been conducted to identify genomic regions associated with *Striga hermonthica* resistance in maize. Genome-wide association study exhibits high mapping resolution and abundant genetic variation due to the high ancestral recombination events in natural populations^35^. Thus, it has been identified as a useful tool for detecting QTNs associated with complex quantitative traits, as well as predicting or identifying causative genes^15^.

Different statistical models have been used for GWAS, the multi-locus model exhibits a higher distinctive power and a lower false-positive rate for detecting QTNs compared with the single-locus GWAS model^18,36^. The adjustments of single-locus GWAS model improves its detection accuracy to an extent, however, the multiple-testing correction (Bonferroni correction) of significance thresholds for single-locus model is too strict. This leads to the exclusion of important loci, especially when large experimental errors occur in field trials of crop genetics. To solve this problem, the application of multi-locus mrMLM is essential. Previous study on GWAS have used the single-locus model to identify quantitative trait nucleotides (QTNs) controlling *Striga* resistance in maize. Adewale et al.^24^ identified 24 SNPs associated with four *Striga* resistance-related traits in early maturing maize inbred lines using single-locus GWAS model. Further annotation analysis identified three putative genes that explained 9 to 42% of the phenotypic variation. The high phenotypic variation explained can be attributed to the single-locus GWAS model used. However, the genomic regions identified by Adewale et al.^24^ differs from those discovered in this study.

In this study, the four multi-locus GWAS models used identified thirty significant SNPs associated the three *Striga* resistance-related traits. This study is the first to use multi-locus GWAS model to identify SNPs associated with *S. hermonthica* resistance in maize. Comparing our GWAS results with previous studies on *S. hermonithica* resistance in maize^9–11^, there were no similar genomic regions detected, however, additional genomic regions associated with *Striga hermonthica* resistance were identified. The annotation analysis identified gene models with potential involvement in plant growth, development, and defense mechanism. Intriguingly, some of the gene models identified encode transcription factors (TFs) including WRKY14, basic helix-loop-helix (bHLH7), bHLH20, basic-domain leucine-zipper (bZIP46), JUNGBRUNNEN 1 and zinc finger proteins. Transcription factors (TFs) are critical regulators of gene expression in all living organisms. They are involved in plant development, cell signaling, and plant defense response.

Studies have shown that most WRKY TFs respond to pathogen attack and act as both positive and negative regulators in complex defense response network^37^. Studies have associated WRKY TFs with *S. hermonthica* resistance mechanism in rice. Swarbrick et al.^38^ reported the up-regulation of genes encoding WRKY TFs in the roots of Nipponbare, a rice variety with resistance to *S. hermonthica*. Also, Mutuku et al.^39^ reported the knockdown of WRKY45 (WRKY45-kd) by RNA interference in rice plants resulted in susceptibility to *S. hermonthica* infestation. bHLH is another TF, they are commonly expressed in response to drought stress and they have been reported in rice^40^. The bHLH family TFs in Populus, PebHLH35 from *Populus euphratica*, has been reported as an essential gene in response to drought by regulating stomatal development and photosynthesis in Arabidopsis^41^.

In addition, the Ring zinc-finger domain superfamily proteins are the most significant TFs known for their finger-like structure and ability to bind to zinc. These proteins have been reported in plants such as wheat (Triticum aestivum), soybean (Glycine max), and rice (Oryza sativa)^42^. Cao et al.^43^ indicated that Ring zinc-finger domain superfamily proteins are involved in resistance to blast fungus infection in rice. The DNA binding with one finger proteins (dofs) also regulate the expression of genes involved in plant development and defense processes^44^. In maize, ZmDof1 has been isolated and connected with C4 photosynthesis^45^, which makes it thrive more than the C3 plants under warmer harsh climates because they are known to be drought resilient. In maize, there are no information on the roles of most of these TFs in *S. hermonthica* resistance; thus, further transcriptomic study will give a better understanding on the role of these TFs in *S. hermonthica* resistance in maize.

Plants generally lack specific cells to defend themselves against attack, but they possess the necessary components for detecting invasion and building up defense response. Xyloglucan endotransglucosylase/hydrolases (XTHs) are cell wall enzymes that are able to graft xyloglucan chains to oligosaccharides^46^. One of its functions in plants is defense reaction against parasitic plants^47^. In tomato, xyloglucan endotransglycosylase/hydrolase (XTH) plays a major role in defense reactions against plant parasite Cuscuta reflexa^47^.

Plants have evolved a series of mechanisms to resist pathogens infection. Most plant disease resistance (R) genes contain nucleotide-binding site (NBS) and leucine-rich repeat (LRR) domains. NBS domains could bind and hydrolyze ATP or GTP, while LRR domains are critical for the formation of protein–protein interactions^48^. NBS-LRR proteins have been suggested as the largest class of known R proteins that can either directly or indirectly recognize the presence of pathogens^49^. R gene proteins are involved in pathogen detection and disease resistance^50^. The Recognition of Peronospora Parasitica 13-like (*RPP13*-like) genes also play important roles in the resistance of various plant diseases including the downy mildew caused by *Peronospora parasitica*. In Arabidopsis, the *RPP13*-Nd, cloned from an ecotype (Niederzenz (Nd-1)), was characterized to resist the infection of various isolates of *P. parasitica*^51^.

Leucine-rich repeat receptor-like protein kinases (LRR-RLKs) are the largest group of receptor-like kinases in plants and play vital roles in development and defense-related processes including cell proliferation, hormone perception, host-specific defense response, wounding response and symbiosis^52^. In Arabidopsis two LRR were identified to regulate cell death and innate immunity^53^. According to Yuan et al.^54^, LRR-RLK can positively regulate plant biotic resistance but negatively regulate plant abiotic tolerance in Arabidopsis . Interestingly, several RLKs were found to possess dual or multiple roles during plant growth and development. For example, ERECTA is involved in both plant development and pathogen defense responses^55^.

U-box proteins significantly contribute to the ability of plants to respond to diverse environmental stresses, due to plant immobility^56^. The ubiquitination pathway regulates growth, development, and stress responses in plants, and the U-box protein family of ubiquitin ligases plays important role in this pathway. In higher plants, U-box-ARM proteins are associated with regulation of cell death and defense^57^. In addition, VQ proteins regulate diverse developmental processes, including responses to biotic and abiotic stresses, seed development, and photomorphogenesis^58^. Members of the VQ family either play a positive or negative role in plant immune response. Plants with loss of function of VQ23 lack resistance to both *Botrytis cinerea* and *Pseudomonas syringae.* While lines, which overexpresses VQ23, showed reduced disease symptoms upon infection with either pathogen^58^.

Cytochrome P450 superfamily proteins were also associated with emerged *Striga* plants, they are often involved in phytoalexin synthesis and the scavenging of toxins. Plants utilize a wide array of cytochrome P450 monoxygenases (P450s) in biosynthetic and detoxification pathways. Several genes encoding P450s were observed to be highly up-regulated during the resistance response to *S. hermonthica* in rice^38^. From this study, it was observed that the genomic regions controlling grain yield under *Striga* infestation differs from the genomic region controlling grain yield under non-infested condition. Genomic regions identified to be associated with plant defense mechanisms will be developed into kompetitive allele-specific PCR (KASP) genotyping assay and this will be validated in independent populations to improve *Striga* resistance breeding in maize before deployment for use in marker-assisted selection. Identified SNPs will also help expedite the use of molecular markers in *Striga* resistance improvement through the use of marker-assisted backcrossing (MABC) to advance the effectiveness of breeding for superior and desirable qualities but susceptible to *Striga* infestation.

In conclusion, most of the significant SNPs discovered in this study encode genes associated with plant defense mechanism. Most of the QTLs identified have not been documented in maize, indicating they are novel and are addition to the already identified QTLs for *Striga* resistance in maize from other studies. QTNs identified in this study can be potentially used to expedite the use of marker-assisted selection (MAS) in breeding for durable resistance to *S. hermonthica* in maize. The chromosomal regions controlling the *Striga* resistance-related traits can also be exploited for selection and effective pyramiding of favorable alleles in *Striga* improvement. Since most of the maize lines used in this study were developed at IITA and have a diverse response to *Striga* infestation, this study will contribute to molecular-marker based transformation of *Striga* resistance breeding in maize.

## Materials and methods

### Genetic materials

A diversity panel of 150 maize inbred lines used in this study was developed by the Maize breeding program of the International Institute of Tropical Agriculture (IITA-Ibadan). The maize inbred lines in this panel were at S_7:9_ stages of inbreeding and had varying reactions to *S. hermonthica* (Supplementary Table S4). Summaries of the genetic backgrounds of source populations of these inbred lines are provided in Table 8. Ten inbred lines with either known resistance (9450), tolerance (5012, 1393, 1368, 4001, 9030, 9071, KU1414-SR, and MMB90) and susceptibility (5057) reactions to *S. hermonthica* were included as benchmarks to assess the performance of the 150 lines**.**

**Table 8:** List of source populations for the 150 inbred lines used in this study.

Source population	Genetic backgrounds of inbred source population	No of lines evaluated
ZDIP	Inbred lines derived from a backcross (BC4) containing a *Zea diploperennis* accession as a donor parent plus lines derived from bi-parental crosses involving one parent derived from the same BC4	39
TZLC	Lines derived from a late-maturing composite formed by crossing TZB-SR with seven field resistant maize inbred lines against *S. hermonthica* plus lines derived from bi-parental crosses involving one parent derived from the same source populations	44
TZEC	Lines developed from an early maturing composite formed by crossing TZESR-W C3 with eight field resistant maize inbred lines against *S. hermonthica* plus lines derived from bi-parental crosses involving one parent derived from the same composite	13
IWDS	Lines extracted from a synthetic formed from medium maturing white inbred lines and improved for resistance to *Striga* and drought plus lines derived from bi-parental crosses involving one parent derived from these synthetic	30
MIXED	Lines derived from diverse source populations plus tolerant lines extensively used as donors of field resistance to form resistance source populations	24

### Phenotypic evaluation and trait measurements

The inbred lines were evaluated under *Striga* infested and non-infested conditions at Abuja (9° 15ʹ N, 7° 20ʹ E; 490 m asl) and Mokwa (9° 21ʹ N, 5° 10ʹ E; 210 m asl) in Nigeria during the main rainy seasons of 2017 and 2018. The experiment was laid out in a 15 X 10 alpha lattice design with two replications planted in a crisscross arrangement. Each experimental unit was planted in adjacent infested and non-infested strips, located opposite each other and separated by a 1.5 m alley. An inbred line was planted within each strip in a 4 m long row, with 0.75 m inter-row spacing and 0.25 m intra-row spacing. Ethylene gas was sprayed two weeks before planting to induce suicidal germination of *Striga* seeds in the soil.

Two maize seeds were planted in a 6 cm deep hole inoculated with 8.5 g of sand mixed with *Striga* seeds. The sand-*Striga* mixture contains approximately 3000 germinable *Striga* seeds. The *Striga* seeds used in this study were collected from sorghum field from the previous planting season in Mokwa and Abuja with farmers’ consent before usage. Two weeks after planting, maize plants were thinned to one plant per hill to attain a population density of 53,333 plant/ha. Fertilizer was applied at the rate of 30 kg/ha of nitrogen, 60 kg/ha each of phosphorus and potassium at planting and an additional 30 kg/ha nitrogen was applied four weeks later. Weeds other than *Striga* were removed from plots manually throughout the planting season. Data were taken under both infested and non-infested conditions, except for *Striga* damage score and *Striga* emergence, recorded only under *Striga* infestation. Data recorded under the two environments included plant height, ear aspect, and grain yield (Supplementary Table S5). Ears were collected separately from each line and shelled to estimate per cent moisture in the grain. Grain yield was then calculated from grain weight adjusted for 15% moisture. This study is geared towards improving IITA maize breeding, and it complies with the country’s local and national regulations.

### Data analysis

#### Phenotypic analysis

Analysis of variance combined across the four year-location, which were hereafter referred to as environments, were computed for all traits measured under *Striga* infested and non-infested conditions based on a mixed-model analysis with restricted maximum likelihood procedure in SAS version 9.4^59^. In this analysis, genotypes were considered fixed while all other factors were random.

Separate analyses were conducted for traits measured under infested and non-infested conditions. The mixed model analysis generated the best linear unbiased estimates (BLUEs), the variance components and broad-sense heritability estimates. In addition, Spearman rank correlations between pairs of environments were computed for the *Striga* resistance-related traits using SAS version 9.4. Also, correlation analysis among the different traits was performed using R software, and results were displayed as heat map.

#### Genotyping and filtering

Genomic DNA was extracted from young leaf samples of the 150 maize inbred lines using the modified cetyltrimethylammonium bromide (CTAB) protocol^60^. Purified DNA was sent to Elshire facility in New Zealand for genotyping-by-sequencing (GBS)^61^. Genomic DNA was digested with the restriction enzyme (*ApeK1*), and genotyping-by-sequencing (GBS) of the libraries were constructed in 96-plex and sequenced on Illumina HiSeq2500 following manufacturer’s protocol. Raw flow cell output was processed to genotype calls using the TASSEL-GBS pipeline. Reads and tags found in each sequencing result were aligned with the *Zea mays* L. genome reference, version AGPV3 (B73 Ref Gen v4 assembly). SNPs with minor allele frequency (MAF) of < 0.05 and missing rate of > 10% were excluded from the genotyping dataset using PLINK 1.9 beta^62^.

#### Population structure and linkage disequilibrium

To explore the genetic relationship among the inbred lines, principal component analysis (PCA) was conducted using factorMiner package in R^63^. The pairwise genetic distance was calculated through identity-by-state (IBS), and the phylogenetic tree was generated using analysis of phylogenetics and evolution (ape) R package^64^. The population stratification among the inbred lines was assessed using Admixture software^65^. The method uses maximum likelihood estimation on data from many loci to estimate individual ancestries among the inbred lines. The analysis was performed using a cross-validation error (k) varying from 2 to 10. The most appropriate *k*-value selected exhibit low cross-validation error compared to other *k*-values. LD among markers was calculated using PLINK software. The window size for LD calculation was set based on the number of SNPs located in the genome. Pairwise linkage disequilibrium was measured using the squared allele frequency correlations, according to Weir,^66^, and assessed by calculating r^2^ for pairs of SNP loci.

#### Marker-trait association analysis

All the phenotypic and genotypic information from the 150 diverse maize inbred lines were used to detect SNPs using four of the GWAS multi-locus models, multi-locus random-SNP-effect MLM (mrMLM), fast multi-locus random-SNP-effect mixed linear model (FASTmrMLM), fast multi-locus random-SNP-effect efficient mixed model association (FASTmrEMMA) and polygenic-background-control-based least angle regression empirical Bayes (pLARmEB), implemented in mrMLM v4.0 (https://cran.r-project.org/web/packages/mrMLM.GUI/index.html). The unified parameter settings for the four methods were as follows; the Q + K model was used, where the population structure value Q was calculated by Admixture software^65^ and the kinship value K was analyzed by the “mrMLM” software package. The limit of detection (LOD) score was set to 6 for robust QTNs for all measured trait. The Manhattan and QQ plots for GWAS were displayed using the R package CMplot.

#### Gene annotation

SNPs detected for *Striga hermonthica* resistance-related traits by the four mrMLM methods were mapped to the maize reference genome B73 RefGen_V4 to identify associated candidate genes. The genes corresponding to each QTN was determined in MaizeGDB according to its physical position. The functional annotations of the candidate genes were predicted in NCBI. The Pairwise LD estimates in the region of interest for significantly associated markers were investigated using Haploview 4.2. Finally, LD plotting was done based on base pairs (bp) distance, using “ggplot2” package in R ^67^.

#### Marker effect prediction and variants comparison

Variants (ref/alt) associated with significant SNPs were identified using *rstatix* package implemented in R, and their effect were compared using ANOVA *p* < 0.05. The nature of the SNP marker (favorable and unfavorable) was defined based on the direct contribution to the traits using *rstatix* and visualized using ggplot2.

## Supplementary Information

Supplementary Figures.

Supplementary Tables.

## Data Availability

Phenotypic data presented are within this document and the genotypic data can be provided upon request.
