# Supplementary material for: Association analysis for resistance to Striga hermonthica in diverse tropical maize inbred lines
Source: Sci Rep. 2021 Dec 17;11:24193. doi: 10.1038/s41598-021-03566-4 (PMC8683441; doi:10.1038/s41598-021-03566-4)
Supplement: Supplementary file 1 — Supplementary Figures. [file 41598_2021_3566_MOESM1_ESM.docx]

**Manuscript title:** ­Association analysis for resistance to *Striga hermonthica* in diverse tropical maize inbred lines

**Authors:** Stanley A.E., Menkir A., Ifie B.E., Paterne A.A., Unachukwu N.N., Meseka S., Mengesha W.A., Bossey B., Kwadwo O., Tongoona P.B., Oladejo O., Sneller C., Gedil M.

**Supplementary Figure S1:** Heatmap showing the correlation among the 3 phenotypic variables under *Striga* infestation; YLDIN_G: Grain yield; STRAT_1= *Striga* rating at 8 WAP; STRAT_2= *Striga* rating at 10 WAP; STRCO_1= emerged *Striga* count at 8 WAP; STRCO_2: emerged *Striga* count at 10 WAP; WAP: weeks after planting.

*, **, ***Significant at *p* ≤ 0.05, *p*  ≤ 0.01 and *p* ≤ 0.001 levels, respectively.


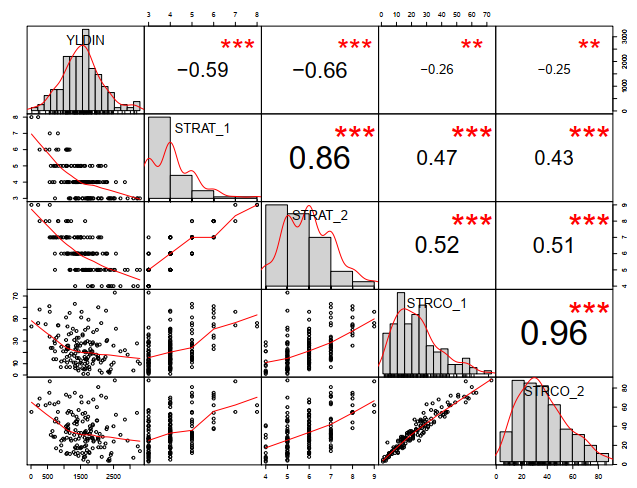


**Supplementary Figure S2**. Genome-wide linkage disequilibrium (LD) decay plot.


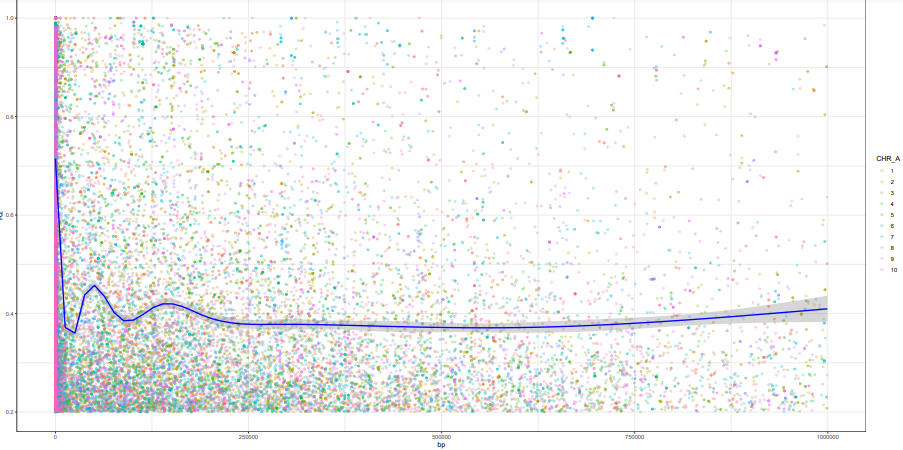


**Supplementary Figure S3.** Heatmap LD haplotype blocks for different SNP markers located on different chromosomes (9 & 10) for grain yield. The R^2^ color key indicates the degree of significant association with the putative genes. The SNP markers are indicated with black color while the associated genes are indicated in blue light


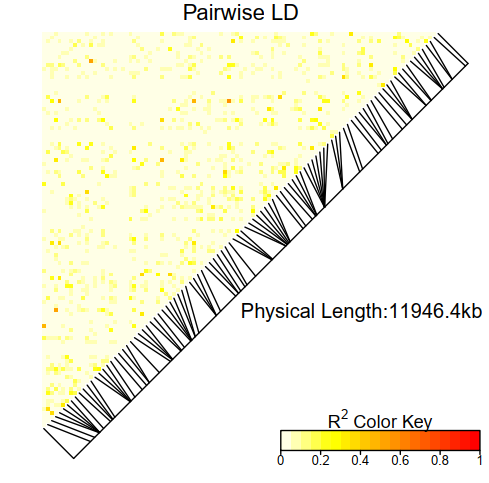


S9_1994432

NLR family CARD protein


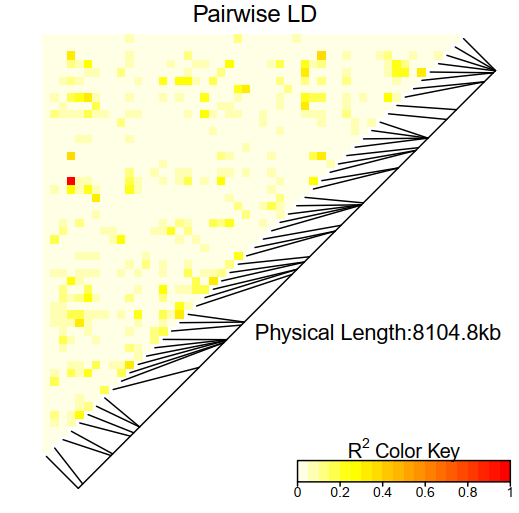


S10_1784894

VQ proteins

U-box domain-containing protein

**Supplementary Figure S4.** Heatmap LD haplotype blocks for different SNP markers located on different chromosomes (5, 7 & 10) for *Striga* damage ratings. The R^2^ color key indicates the degree of significant association with the putative genes. The SNP markers are indicated with black color while the associated genes are indicated in blue light


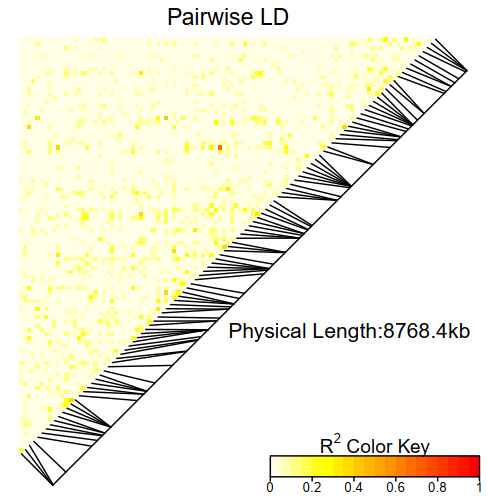


S7_10795659


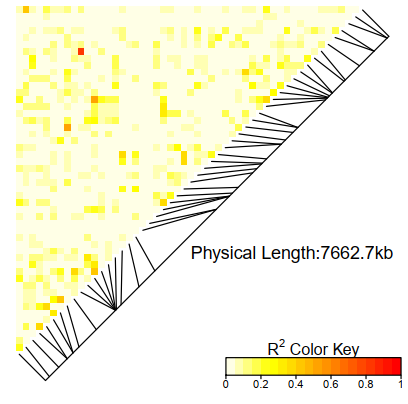


S10_2743583


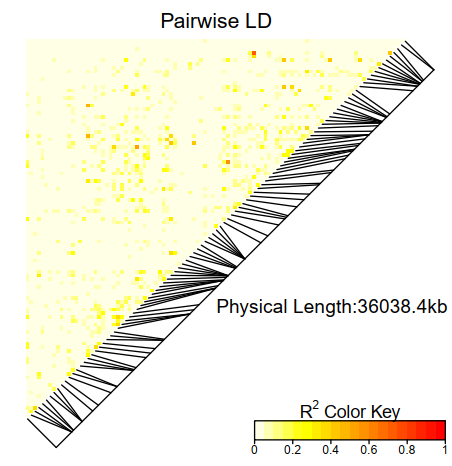


S5_70442824

Transcription factor bHLH7

uncharacterized LOC100381459

leucine-rich repeat extensin-like protein 3

putative cytochrome P450 superfamily protein

**Supplementary Figure S5.** Heatmap LD haplotype blocks for different SNP markers located on different chromosomes (3 & 9) for emerged *Striga* plants. The R^2^ color key indicates the degree of significant association with the putative genes. The SNP markers are indicated with black color while the associated genes are indicated in blue light


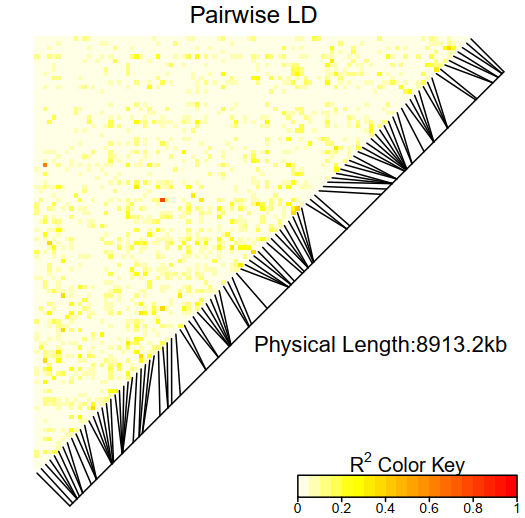


S9_7727167


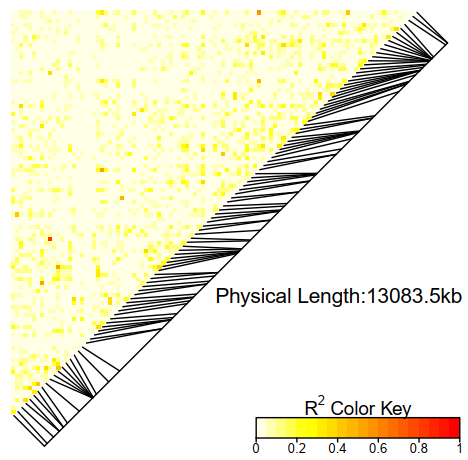


S3_74335447

bZIP transcription factor 46

uncharacterized LOC100382572
