# Supplementary material for: Association analysis for resistance to Striga hermonthica in diverse tropical maize inbred lines
Source: Sci Rep. 2021 Dec 17;11:24193. doi: 10.1038/s41598-021-03566-4 (PMC8683441; doi:10.1038/s41598-021-03566-4)
Supplement: Supplementary file 2 — Supplementary Tables. [file 41598_2021_3566_MOESM2_ESM.docx]

**Manuscript title:** ­Association analysis for resistance to *Striga hermonthica* in diverse tropical maize inbred lines

**Authors:** Stanley A.E., Menkir A., Ifie B.E., Paterne A.A., Unachukwu N.N., Meseka S., Mengesha W.A., Bossey B., Kwadwo O., Tongoona P.B., Oladejo O., Sneller C., Gedil M.

**Supplementary Table S1:** Spearman’s rank correlation between means of maize inbred lines evaluated at four test environments. Env= environments, WAP= weeks after planting, **** = significant at p ≤ 0.0001. Env1=Abuja 2017, Env2= Abuja 2018, Env3= Mokwa 2017, Env4= Mokwa 2018

|  | **Grain yield under *Striga* infestation** | | |
| --- | --- | --- | --- |
|  | **ENV2** | **ENV3** | **ENV4** |
| **ENV1** | 0.56**** | 0.55**** | 0.48**** |
| **ENV2** |  | 0.45**** | 0.52**** |
| **ENV3** |  |  | 0.50**** |
|  | **Grain yield under non-infested condition** | | |
|  | **ENV2** | **ENV3** | **ENV4** |
| **ENV1** | 0.57**** | 0.44**** | 0.53**** |
| **ENV2** |  | 0.40**** | 0.59**** |
| **ENV3** |  |  | 0.44**** |
|  | ***Striga* damage rating at 8 WAP** | | |
|  | **ENV2** | **ENV3** | **ENV4** |
| **ENV1** | 0.47**** | 0.60**** | 0.35**** |
| **ENV2** |  | 0.50**** | 0.38**** |
| **ENV3** |  |  | 0.36**** |
|  | ***Striga* damage rating at 10 WAP** | | |
|  | **ENV2** | **ENV3** | **ENV4** |
| **ENV1** | 0.63**** | 0.68**** | 0.44**** |
| **ENV2** |  | 0.57**** | 0.52**** |
| **ENV3** |  |  | 0.44**** |
|  | **Emerged *Striga* plants at 8 WAP** | | |
|  | **ENV2** | **ENV3** | **ENV4** |
| **ENV1** | 0.70**** | 0.55**** | 0.63**** |
| **ENV2** |  | 0.57**** | 0.61**** |
| **ENV3** |  |  | 0.58**** |
|  | **Emerged *Striga* plants at 10 WAP** | | |
|  | **ENV2** | **ENV3** | **ENV4** |
| **ENV1** | 0.70**** | 0.52**** | 0.62**** |
| **ENV2** |  | 0.60**** | 0.65**** |
| **ENV3** |  |  | 0.54**** |

**Supplementary Table S2:** Mean, range and Standard error of agronomic traits under *Striga* and non-infested conditions across test environments

| Variables | Minimum | Maximum | Mean | Std error | Heritability (%) |
| --- | --- | --- | --- | --- | --- |
| **Infested** |  |  |  |  |  |
| Grain yield (kg/ha) | 13 | 3299.25 | 1580 | 255 | 82 |
| *Striga* damage rating 8 WAP | 3 | 8 | 4 | 0.4 | 84 |
| *Striga* damage rating 10 WAP | 4 | 9 | 6 | 0.43 | 84 |
| Emerged *Striga* plants 8 WAP | 1 | 73 | 23 | 5.85 | 85 |
| Emerged *Striga* plants 10 WAP | 3 | 88 | 35 | 8.28 | 81 |
| **Non-infested** | |  |  |  |  |
| Grain yield (kg/ha) | 706 | 4171 | 2098 | 437.27 | 80 |

**Supplementary Table S3**. LD decay distance at R2 < 0.2 on the different maize chromosomes and the entire maize genome.

| **Chromosome** | **Size (Mbp)** | **Mean R^2^** | **LD decay distance (kb) at R^2^ < 0.2** |
| --- | --- | --- | --- |
| 1 | 3.08 | 0.03 | 2891.53 |
| 2 | 2.98 | 0.04 | 2713.76 |
| 3 | 3.35 | 0.04 | 3041.05 |
| 4 | 3.59 | 0.04 | 3299.72 |
| 5 | 3.08 | 0.04 | 2796.40 |
| 6 | 2.73 | 0.04 | 3064.51 |
| 7 | 3.35 | 0.04 | 3026.76 |
| 8 | 3.62 | 0.04 | 3305.17 |
| 9 | 3.4 | 0.04 | 3151.39 |
| 10 | 3.19 | 0.04 | 3059.33 |
| Whole genome | 3.21 | 0.04 | 3002.16 |
| Average | 3.24 | 0.04 | 3034.96 |

**Supplementary Table S4:** List of diverse maize inbred lines used for GWAS

| ENTRY | Group | NAME |
| --- | --- | --- |
| 1 | TZLC | TZLCompIC4S1-50-3-B-3-B*7 |
| 2 | TZLC | (ACRSYN-W-S2-173-B*4/TZLCompIC4S1-37-5-BBB)-3-B*4 |
| 3 | TZLC | (ACRSYN-W-S2-173-B*4/TZLCompIC4S1-37-5-BBB)-25-B*4 |
| 4 | TZLC | (ACRSYN-W-S2-173-B*4/TZLCompIC4S1-37-1-B*4)-54-B*4 |
| 5 | TZLC | (ACRSYN-W-S2-173-B*4/TZLCompIC4S1-37-1-B*4)-17-B*4-B |
| 6 | ZDIP | ZdiploBC4-472-2-3-4-3-B-3-B*8 |
| 7 | ZDIP | (1393/ZDiploBC4-19-4-1-#-3-1-B-1-B*4)-2-B*4 |
| 8 | ZDIP | (1393/ZDiploBC4-19-4-1-#-3-1-B-1-B*4)-3-B*4 |
| 9 | ZDIP | (1393/ZDiploBC4-19-4-1-#-3-1-B-1-B*4)-43-B*4 |
| 10 | ZDIP | (1393/ZDiploBC4-19-4-1-#-3-1-B-1-B*4)-46-B*4 |
| 11 | ZDIP | (1393/ZDiploBC4-19-4-1-#-3-1-B-1-B*4)-61-1-1-BB-B |
| 12 | ZDIP | (ZDiploBC4-472-2-2-1-2-3-B-1-B*5/ZDiploBC4-19-4-1-#-3-1-B-1-B*4)-43-1-BB-B |
| 13 | ZDIP | (ZDiploBC4-472-2-2-1-2-3-B-1-B*5/ZDiploBC4-19-4-1-#-3-1-B-1-B*4)-44-1-BB-B |
| 14 | ZDIP | (ZDiploBC4-472-2-2-1-2-3-B-1-B*5/ZDiploBC4-19-4-1-#-3-1-B-1-B*4)-34-1-BB-B |
| 15 | IWDS | IWD-SYN-STR-C3-1-1-B*5 |
| 16 | IWDS | IWD-SYN-STR-C3-1-2-B*5 |
| 17 | IWDS | IWD-SYN-STR-C3-11-1-B*5 |
| 18 | IWDS | IWD-SYN-STR-C3-14-1-B*5 |
| 19 | IWDS | IWD-SYN-STR-C3-26-1-B*5 |
| 20 | IWDS | IWD-SYN-STR-C3-32-2-B*5 |
| 21 | IWDS | IWD-SYN-STR-C3-40-1-B*5 |
| 22 | IWDS | IWD-SYN-STR-C3-47-1-B*5 |
| 23 | IWDS | IWD-SYN-STR-C3-52-1-B*5 |
| 24 | IWDS | IWD-SYN-STR-C3-52-4-B*5 |
| 25 | IWDS | IWD-SYN-STR-C3-53-2-B*5 |
| 26 | IWDS | IWD-SYN-STR-C3-55-3-B*5 |
| 27 | IWDS | IWD-SYN-STR-C3-56-4-B*5 |
| 28 | MIXED | 5012 |
| 29 | IWDS | IWD-SYN-STR-C3-50-2-B-B-B-B |
| 30 | MIXED | STR Low Emerg. Pool CO S3 670-1-5-3-3-B-1-B*9 |
| 31 | IWDS | IWD-SYN-STR-C3-18-1-B-B-B |
| 32 | IWDS | IWD-SYN-STR-C3-46-5-B-B-B |
| 33 | IWDS | IWD-SYN-STR-C3-52-2-B-B-B |
| 34 | MIXED | (9450xCM 116x9450)-3-3-1-2-1-B-B-B-B-B-B-B-B |
| 35 | TZLC | (ACR97SYN-Y-S1-24-B*4/ACR97TZLComp1-YS155-4-1-3-B*4)-13-1-BB-B |
| 36 | TZLC | (ACR97SYN-Y-S1-79-B*4/ACR97TZLComp1-YS155-4-1-3-B*4)-19-1-BB-B |
| 37 | TZLC | (ACR97SYN-Y-S1-79-B*4/ACR97TZLComp1-YS155-4-1-3-B*4)-24-1-BB-B |
| 38 | TZLC | (ACR97TZLComp1-YS155-4-1-3-B*4/ACR97SYN-Y-S1-76-B*4)-1-1-B-B |
| 39 | TZLC | (ACR97TZLComp1-YS155-4-1-3-B*4/ACR97SYN-Y-S1-76-B*4)-11-1-BB-B |
| 40 | TZLC | (ACR97SYN-Y-S1-24-B*4/ACR97TZLComp1-YS155-4-1-3-B*4)-30-1-BB-B |

| ENTRY | Group | NAME |
| --- | --- | --- |
| 41 | TZEC | (TZECOMP5-Y-C7-S3-56-B*4/TZECOMP5-25-1-1-3-#-2-B*4)-28-1-BB-B |
| 42 | TZEC | (TZECOMP5-Y-C7-S3-56-B*4/TZECOMP5-25-1-1-3-#-2-B*4)-36-1-BB-B |
| 43 | TZEC | (TZECOMP5-Y-C7-S3-56-B*4/TZECOMP5-25-1-1-3-#-2-B*4)-43-1-BB-B |
| 44 | TZEC | (TZECOMP5-Y-C7-S3-150-B*4/TZECOMP5-25-1-1-3-#-2-B*4)-9-1-BB-B |
| 45 | TZEC | (TZECOMP5-Y-C7-S3-150-B*4/TZECOMP5-25-1-1-3-#-2-B*4)-10-1-BB-B |
| 46 | TZEC | (TZECOMP5-Y-C7-S3-150-B*4/TZECOMP5-Y-C7-S3-56-B*4)-65-1-BB-B |
| 47 | TZEC | (TZECOMP5-Y-C7-S3-150-B*4/TZECOMP5-Y-C7-S3-56-B*4)-42-1-BB-B |
| 48 | TZLC | (KU1409/DE3/KU1409)S2-27-B*4/ACR97TZL-CCOMP1-Y-S3-40-3-B*6-6-B-1-B |
| 49 | TZLC | (KU1409/DE3/KU1409)S2-18-2-BBB/ACR97TZL-CCOMP1-Y-S3-35-2-B*8-12-2-1-B |
| 50 | TZLC | (KU1409/DE3/KU1409)S2-18-2-BBB/ACR97TZL-CCOMP1-Y-S3-35-2-B*8-82-3-3-B |
| 51 | TZLC | (KU1409/DE3/KU1409)S2-18-2-BBB/ACR97TZL-CCOMP1-Y-S3-35-2-B*8-40-B-1-B |
| 52 | TZLC | (KU1409/DE3/KU1409)S2-6-B*4/ACR97TZL-CCOMP1-Y-S3-33-6-B*6-32-2-1-B |
| 53 | ZDIP | (ZDiploBC4-19-4-1-#-3-1-B-1-BxPioneerIRxZDiploBC4-19-4-1-#-3-1-B-1-B)-4-1-2-1-B*9 |
| 54 | MIXED | 9450xPioneerIRx9450BC1-1-23-1-4-B*10 |
| 55 | ZDIP | (ZDiploBC4-19-4-1-#-3-1-B-1-BxPioneerIRxZDiploBC4-19-4-1-#-3-1-B-1-B)-4-2-1-6-B*9 |
| 56 | ZDIP | (CML181/ZdiploBC4-472-2-3-4-3-B-3-BBB)-38-B*11 |
| 57 | ZDIP | (CML181/ZdiploBC4-472-2-2-1-2-3-B*5)-23-B*11 |
| 58 | ZDIP | (CML181/ZDiploBC4-472-2-2-1-6-4-B-1-B*5)-15-B*11 |
| 59 | MIXED | 9071STR |
| 60 | TZLC | TZL Comp. IC4 S1-38-5-B-B-B |
| 61 | ZDIP | Z.diplo.BC4-472-2-2-1-2-3-B-B-B-B-B |
| 62 | ZDIP | Z.diplo.BC4-376-1-1-#-3-1-B-2-B-B |
| 63 | ZDIP | Z.Diplo.BC4-472-2-3-1-1-B-1-B-B-B-B-B |
| 64 | ZDIP | Z. Diplo.BC4-472-2-1-1-2-1-B-1-B-B-B-B |
| 65 | TZLC | ACR97SYN-Y-S1-24-B-B-B |
| 66 | MIXED | SYN-W-S2-99-B-B-B |
| 67 | TZEC | TZE COMP5-25-1-1-3-#-2-B-B-B |
| 68 | TZEC | TZEComp.5-Y-20-1-1-3-#-2-B-B-B-B |
| 69 | TZEC | TZECOMP5-Y-C7-S3-55-B-B-B |
| 70 | TZEC | TZECOMP5-Y-C7-S3-56-B-B-B |
| 71 | TZEC | TZE Comp.5-Y-21-1-1-2-#-B-B-B-B |
| 72 | TZEC | TZECOMP5-Y-C7-S3-150-B-B-B |
| 73 | ZDIP | ZDiploBC4-467-4-1-2-1-1-B-1-B*6 |
| 74 | ZDIP | ZdiploBC4-472-2-3-4-3-B-2-B*8 |
| 75 | TZLC | ACRSYN-W-S2-173-B*7 |
| 76 | MIXED | Syn-Y-STR-(43-2)-1-1-5-1-B*6 |
| 77 | TZLC | ACR97TZL-CCOMP1-Y-S3-34-2-B*9 |
| 78 | ZDIP | ZeaDiploBC4-WC3-74-1-3-B*7 |
| 79 | MIXED | 5057xB73LPAx5057-30-1-4-B*8 |
| 80 | MIXED | STRLowEmergPoolCOS3670-1-5-3-3-B-1-B*11 |

| ENTRY | Group | NAME |
| --- | --- | --- |
| 81 | TZLC | ACR97TZLComp1-YS129-2-2-1-B-2-B*11 |
| 82 | MIXED | 1368STR |
| 83 | MIXED | 1393-BBB |
| 84 | TZLC | ACR97TZL-CCOMP1-Y-S3-12-2-B*8 |
| 85 | TZLC | ACR97TZL-CCOMP1-Y-S3-6-1-B*9 |
| 86 | TZLC | ACR97TZL-CCOMP1-Y-S3-35-2-B*10 |
| 87 | MIXED | 9450xKI21-3-2-2-2-1-B*8 |
| 88 | TZLC | (TZLCOMP1-WC6/(WhiteDTSTRSyn/TZLCOMP1-W))-165-BBB-1 |
| 89 | TZLC | (TZLCOMP1-WC6/(WhiteDTSTRSyn/TZLCOMP1-W))-259-BBB-3 |
| 90 | MIXED | 1368 |
| 91 | MIXED | 4001 |
| 92 | MIXED | 9030 |
| 93 | MIXED | 9071 |
| 94 | MIXED | 9450 |
| 95 | MIXED | 4001 STR |
| 96 | MIXED | 9030 STR |
| 97 | TZLC | ACR97SYN-Y-S1-79-B-B-B-B/Z. Diplo.BC4-467-4-1-2-1-1-B-1-B-B-B-B-B |
| 98 | TZLC | ACR97TZL-CCOMP1-Y-S3-56-1-B-B |
| 99 | MIXED | KU1414-SR |
| 100 | MIXED | MMB90 |
| 101 | MIXED | POP43 SR S5-3-1-1-1-1-1-B-B-B-B-B |
| 102 | TZLC | TZL Comp. IC4 S1-37-1-B-B-B-B-B-B |
| 103 | TZLC | TZL Comp. IC4 S1-37-5-B-B-B-B-B-B |
| 104 | TZLC | TZLComp1(TC87)-2-#-4-1-4-B-1-B*7 |
| 105 | ZDIP | Z. Diplo.BC4-467-4-1-2-1-1-B-1-B-B-B-B-B-B |
| 106 | ZDIP | ZDiploBC4-551-2-1-#-1-3-B-2-B*7 |
| 107 | TZLC | ACR97SYN-Y-S1-38-B-B |
| 108 | TZLC | ACR97SYN-Y-S1-79-B-B-B |
| 109 | TZLC | ACR97TZL-CCOMP1-Y-S3-33-6-B*8 |
| 110 | MIXED | KU1414 STR |
| 111 | TZLC | TZLComp1-(TC87)-2-#-5-1-5-B-1-B*9 |
| 112 | ZDIP | Z. Diplo. BC4-282-5-2-2-1-B-1-B-B-B |
| 113 | ZDIP | Z. diplo. BC4-472-2-2-1-6-4-B-B |
| 114 | ZDIP | Z. Diplo.BC4 -290-4-2-1-1-B-1-B-B-B |
| 115 | ZDIP | Z. Diplo.BC4-472-2-2-1-2-3-B-1-B-B-B |
| 116 | ZDIP | Z.Diplo.BC4-19-4-1-#-3-1-B-1-B-B-B-B-B-B |
| 117 | ZDIP | Z.diplo.BC4-290 |
| 118 | ZDIP | Z.diplo.BC4-472 |
| 119 | MIXED | 5057 |
| 120 | TZLC | (TZLComp. 1 C6-W-11-1-2)-B-B |

| ENTRY | Group | NAME |
| --- | --- | --- |
| 121 | TZLC | (TZLComp. 1 C6-W-16-1-1)-B-B |
| 122 | TZLC | (TZLComp. 1 C6-W-34-1-1)-B-B |
| 123 | TZLC | (TZLComp. 1 C6-W-39-1-1)-B-B |
| 124 | TZLC | (TZLComp. 1 C6-W-44-1-1)-B-B |
| 125 | TZLC | (TZLComp. 1 C6-W-50-1-1)-B-B |
| 126 | TZLC | (TZLComp. 1 C6-W-82-1-3)-B-B |
| 127 | TZLC | (TZLComp. 1 C6-W-143-1-1)-B-B |
| 128 | TZLC | (((TZL COMP1-W C6*2/(White DT STR Syn))-DT C1-6-1-1)-B-B |
| 129 | IWDS | (((IWD C3 SYN*2/(White DT STR Syn))-DT C1 -21-1-2)-B-B |
| 130 | IWDS | (((IWD C3 SYN*2/(White DT STR Syn))-DT C1 -135-1-2)-B-B |
| 131 | IWDS | (((IWD C3 SYN*2/(White DT STR Syn))-DT C1 -168-1-2)-B-B |
| 132 | IWDS | (((IWD C3 SYN*2/(White DT STR Syn))-DT C1 -169-1-1)-B-B |
| 133 | IWDS | (((IWD C3 SYN*2/(White DT STR Syn))-DT C1 -170-1-1)-B-B |
| 134 | IWDS | (((IWD C3 SYN*2/(White DT STR Syn))-DT C1 -170-1-2)-B-B |
| 135 | IWDS | (((IWD C3 SYN*2/(White DT STR Syn))-DT C1 -173-1-1)-B-B |
| 136 | IWDS | (((IWD C3 SYN*2/(White DT STR Syn))-DT C1 -173-1-2)-B-B |
| 137 | IWDS | (((IWD C3 SYN*2/(White DT STR Syn))-DT C1 -178-1-1)-B-B |
| 138 | IWDS | (((IWD C3 SYN*2/(White DT STR Syn))-DT C1 -181-1-2)-B-B |
| 139 | IWDS | (((IWD C3 SYN*2/(White DT STR Syn))-DT C1 -181-1-4)-B-B |
| 140 | IWDS | (((IWD C3 SYN*2/(White DT STR Syn))-DT C1 -193-1-2)-B-B |
| 141 | IWDS | (((IWD C3 SYN*2/(White DT STR Syn))-DT C1 -193-1-3)-B-B |
| 142 | ZDIP | (Z. Diplo.BC4C3-W-DT C1-31-1-2)-B-B |
| 143 | ZDIP | (Z. Diplo.BC4C3-W-DT C1-33-1-1)-B-B |
| 144 | ZDIP | (Z. Diplo.BC4C3-W-DT C1-43-1-1)-B-B |
| 145 | ZDIP | (Z. Diplo.BC4C3-W-DT C1-46-1-1)-B-B |
| 146 | ZDIP | (Z. Diplo.BC4C3-W-DT C1-57-1-1)-B-B |
| 147 | ZDIP | (Z. Diplo.BC4C3-W-DT C1-62-1-2)-B-B |
| 148 | ZDIP | (Z. Diplo.BC4C3-W-DT C1-64-1-1)-B-B |
| 149 | ZDIP | (Z. Diplo.BC4C3-W-DT C1-77-1-1)-B-B |
| 150 | ZDIP | (Z. Diplo.BC4C3-W-DT C1-98-1-1)-B-B |

**Supplementary Table S5**. Traits measured, method of evaluation and their unit

| S/N | Traits | Traits Abb. | Trait description | Unit |
| --- | --- | --- | --- | --- |
| 1 | Emerged Striga plant | STRCO 1 & 2 | Number of emerged striga plant per plot at 8 and 10 weeks after planting [WAP] | Count |
| 2 | Striga damage rating | STRRAT 1 & 2 | It is scored on a scale of 1-9, where 1= no visible host plant damage symptom and 9 = all leaves are completely scorched and finally dead plants. Taken at 8 and 10 WAP | Scale |
| 3 | Grain yield | YLDIN | Calculated from ear weight and grain moisture | kg/ha |
